# Supplementary material for: Spatio-temporal control of mutualism in legumes helps spread symbiotic nitrogen fixation
Source: eLife. 2017 Oct 12;6:e28683. doi: 10.7554/eLife.28683 (PMC5687860; doi:10.7554/eLife.28683)
Supplement: Supplementary file 1. [file elife-28683-supp1.docx]

**Supplementary File 1.** Primers used in this study.

| Primer name | Primer sequence 5'-3' | Use |
| --- | --- | --- |
| oCBM1821 | CGGGATCCGATCACACCCAACGATGAGA | Amplification of the *nifH* upstream region |
| oCBM2362 | GCTCTAGAGTTGCCAAGCGACGTATTT | Amplification of the *nifH* upstream region |
| oCBM1822 | CGGGATCCGTGCAGATCGACGAGTCAAA | Amplification of the *nifH* downstream region |
| oCBM2363 | CCGGAATTCGAGTACTGGCCACAGCCAAC | Amplification of the *nifH* downstream region |
| oCBM1824 | ACGAAATTGTCCGATGGCTA | Verification of the *nifH* deletion |
| oCBM1825 | TAGTAATTGCGACGCGATCC | Verification of the *nifH* deletion |
| oCBM2619 | TCTAGAGTGGTGGTGATCTCGCAATC | Amplification of the *glmS*-Ralta_A206 upstream region |
| oCBM2620 | GGTACCCCTAGGGTGAGGCAAGGCGTTCAG | Amplification of the *glmS*-Ralta_A0206 upstream region |
| oCBM2621 | GGTACCACTAGTCTGAACGCCTTGCCTCAC | Amplification of the *glmS*-Ralta_A0206 downstream region |
| oCBM2622 | GAATTCCAGCTGCATCACTTCGTCAC | Amplification of the *glmS*-Ralta_A0206 downstream region |
